# Railway security checks at the border: between intrusive security technologies and fundamental traveller rights

**DOI:** 10.12688/openreseurope.17727.2

**Published:** 2024-10-15

**Authors:** Kacper Kubrak, Grigore M. Havârneanu

**Affiliations:** 1 Security Division, International Union of Railways (UIC), 16 rue Jean Rey, Paris, Île-de-France, 75015, France

**Keywords:** border security, railway, EU, securitization, migrant flow

## Abstract

European railway borders are facing a particular exposure to security threats and need a delicate balance between securitization and free movement, especially amid globalisation, the current geopolitical landscape and increased migrant flows. For example, the war in Ukraine illustrated the challenges experienced at the Eastern EU borders by the refugee migration surge in early 2022. This exploratory focuses on the European border security control process from the rail border perspective. It encompasses a descriptive synthesis of the lessons learned from the UIC Refugee Task Force as well as insights from the ongoing EU-funded Horizon Europe project ODYSSEUS (Unobtrusive Technologies for Secure and Seamless Border Crossing for Travel Facilitation). Project ODYSSEUS aims to support the security and integrity of the European space, reduce illegal movements of people and goods across EU borders, facilitate travelling for citizens all while protecting fundamental rights of travellers. The project will test a combination of multi-behavioural and General Data Protection Regulation (GDPR) compliant biometric user identity verification tools, allowing, when possible, citizens to cross EU border without any interruption or queue. Further, novel luggage and baggage checks will allow citizens’ vehicles and cargos to be remotely checked at land borders to speed up the border check processes in a secure and reliable manner. The project will run three pilot tests at road, rail and water borders. In this paper, we analyse the implementation of project’s technologies in the rail border crossing pilot test and discuss the implications for the actors involved in the process of railway border crossing (e.g., border authorities, railway operators and railway travellers).

## Plain language summary

European railway borders are facing a particular exposure to security threats such as irregular migration, document fraud, organized crime, or refugee migration surges. These can pose significant challenges to EU border security. This paper will focus on the European border security control process from the rail border perspective offering insights from the ongoing EU-funded project ODYSSEUS (Unobtrusive Technologies for Secure and Seamless Border Crossing for Travel Facilitation). This project aims to support the security and integrity of the European space, reduce illegal movements of people and goods across EU borders, facilitate travelling for citizens all while protecting fundamental rights of travellers. The project will run three pilot tests at road, rail and water borders. In this paper, we will analyse the implementation of project’s technologies in the passenger rail border crossing test which will help citizens to cross EU borders without any interruption or queue. We will also discuss the implications for the actors involved in the process of railway border crossing (e.g., border authorities, railway operators and railway travellers).

## Introduction

The external borders of the European Union (EU) have become increasingly challenged by the dynamic migrant flows engendered by the ever-evolving geopolitical landscape. More than 331,553 attempts of illegal border-crossing at the EU’s external borders were reported by the Member States in the year 2022 ( Frontex, 2022). This number excludes the refugee flow from Ukraine, which is reported to exceed 4.3 million non-EU citizens since the beginning of February 2022 ( European Commission, 2023a). Moreover, the issues faced at the external borders of the EU extend beyond the regular migration challenges. Irregular migration, document fraud, organized crime, such as drugs smuggling, trafficking of firearms or of human beings, all pose a significant threat to EU border security. In addressing these challenges, it is crucial to ensure that migrants and refugees are not left in a vulnerable state, similar to *Homo Sacer (* Nikolopoulou *et al.,* 2000), a figure in Roman law without legal protections. Transposed into the modern-day context of EU cross-border travel, it refers to individuals who may be excluded from fundamental EU law protection and may be subject to arbitrary violence. European institutions acknowledge that, there are risks and threats to which passengers are exposed during cross-border travel which include but are not limited to security risks, potential criminal activities, disruptions to transportation services, health and safety concerns and instances of discrimination ( Frontex, 2023). Recognizing and understanding these is essential for devising comprehensive strategies to safeguard passengers' well-being and ensure a secure and comfortable travel experience for all travellers, including non-EU citizens.

## Aim and structure of the paper

This descriptive paper focuses on the European border security control process from the rail border perspective. The scope of the current article is limited to a critical reflection on security challenges linked to the European railway border crossing context and the passenger train pilot test planned in the ODYSSEUS project. It aims to explore the aspects of border securitization in the context of train border crossing operations, the potential implications of disruptive technologies in border control, emphasizing their possible impacts on the rights of passengers involved in these operations.

## Challenges in the European border management

The growing number of people crossing EU external borders, the size and complexity of EU cross-border travel, coupled with various socio-economic crises, has led to the perception of people flows on the border as a significant challenge, especially to EU countries located on the eastern bank of the Schengen area. According to EU statistics of overall travel within the EU in 2018, 83% of the journeys were made by car, 9% by bus, and 8% by train ( European Commission, 2020a), and these proportions have remained relatively consistent over the past years ( Eurostat, 2024). Even though it may seem that railway transport only serves as a fraction of overall travel of passengers in Europe, it still plays a critical role in the integrity of the continent’s infrastructure. Europe’s 2015 migration crisis may serve as an exemplary case. That year alone, 1,255,600 first time asylum seekers applied for international protection in the Member States of the EU ( Eurostat, 2016). Even though the immigration mostly took place via land vehicles or maritime transport, railway industry was still heavily affected. For example, Deutsche Bahn (2015) reported that from September to November that year, 270,000 illegal entries at the railway premises were reported and more than 170,000 refugees were transported in special trains. Similarly, a study performed in the EU BODEGA project revealed that the Greek Boder Authorities had to close the railway border crossing point from Idomeni which had become unmanageable because of the very high number of migrants arriving by train daily during this same period ( Le Guellec *et al.*, 2018). Five years later, Polish State Railway (2023) reported that during the first year of the refugee crisis in Ukraine there were more than 2,7 million passengers transported on the Polish – Ukrainian border.

In this context, it is also important to underline that the cross-border challenges caused by infrastructure capacities and logistics are further compounded by the political debates within the EU, especially after the 2015 migration crisis. For example, policies like the “Return Directive” ( European Parliamentary Research Service, 2020), which provide a framework for the return of irregular migrants to their home countries, may be perceived as a tool of territorial control and diffusion ( Harsha, 2021). Such migrants may feel pressured to leave the EU despite the presumed "voluntariness" of the directive. They may also perceive that the legal protection available in the appeal process is insufficient. Furthermore, EU border externalization strategies, which involve the management of migration flows, can intensify these concerns by creating barriers that complicate migrants access to fair and humane treatment - for example, by blocking certain migration routes over land and sea, therefore preventing migrants from reaching asylum ( Harsha, 2021). Additionally, socio-economical challenges within Europe, and socio-demographic challenges which piled-up after the past migration crises may have stimulated anti-democratic notions within some European states; this may gain traction by exploiting fears around economic insecurity coming from the migration, fueling racism and anti-immigrant sentiments ( Brown, 2019).

Therefore, while technological advancements and improvements in transport infrastructure aim to facilitate cross-border travel, political currents and the societal *Dispositif* ( Foucault, 1994)—a system of control that includes technologies, legal frameworks, and state practices based on fear—often deem migrants as an inconvenience and security threat. This Dispositif tends to focus on excluding what it deems "abnormal" ( Bigo & Tsoukala, 2008) which can complicate the management of migration flows.

Considering these aspects and the fluctuation of migrant flows throughout EU borders, there is an increasing demand for technological advancements in border crossing systems. The aim of these technologies would be to address challenges posed by the intricate external border dynamics while taking into account societal concerns about EU internal security. Furthermore, to complement these advancements, EU policies aim to create a more streamlined and secure control process at external borders for Third Country Nationals (TCNs), and a more seamless and fluid travel experience for EU citizens, while also facilitating the job of Border Guard Authorities (BGA) ( European Commission, 2020b).

While these technologies must be robust and effective, there is a risk that excessive, constant, and invisible surveillance could transform these systems into modern-day *Panopticon* ( Bentham, 1995), a metaphor derived from Bentham’s prison design, where individuals are constantly surveilled without their knowing. Therefore, it is crucial that these new systems also adhere to legal and fundamental rights of travellers, ensuring sufficient balance between ethics and protection from potential threats.

EU Horizon Europe project ODYSSEUS (Unobtrusive Technologies for Secure and Seamless Border Crossing for Travel Facilitation) serves as a part of the above-mentioned efforts. The project seeks to optimize the border crossing experience for both travellers and relevant BGAs, while safeguarding the fundamental rights of passengers that wish to enter European space by land, water and air. Concurrently, ODYSSEUS aims to facilitate border crossing by implementing and testing the means of identification methods involving multi-behavioural and biometric mechanisms, that will be integrated as a mobile cross-border platform that can be accessed by travellers. As a part of the project’s endeavours, a remote luggage and baggage inspection system will also be tested. The overarching objective of the project is to contribute to a seamless and uninterrupted passenger transit without the need for long halts, thereby maintaining operational speed and efficiency of overall cross-border travel process.

ODYSSEUS started in January 2023 and will run until December 2025. It involves the active participation of 14 partners from 12 European countries (ten EU Member States and two associated countries: the UK and the Republic of Moldova). The consortium partners include global technology leaders, small and medium enterprises, end-user bodies (including four BGAs) and one research institute. The International Union of Railways (Union Internationale Chemins de Fer – UIC) is the consortium partner which acts as an overarching representative of rail end-users. UIC, founded in 1922 in Paris, is the worldwide railway association which facilitates the exchange of knowledge, best practices, and technological advancements in the railway sector. The organization consists of 218 worldwide carriers, infrastructure managers and railway-oriented firms. UIC's role in the ODYSSEUS project involves collecting the needs and requirements of the railway sector with respect to border security and defining the rail use cases. UIC will also lead the pilot at the train border with a particular focus on the seamless passenger identity verification and the execution of a train border pilot trial. The pilot planned for the first quarter of 2025 will evaluate the practical application and functionality of the technologies within the context of train border crossings.

## ODYSSEUS train pilot trials

Taking into consideration the current stage of the ODYSSEUS project, the key objectives applicable for the train pilot project consists of:

1) Facilitating BGAs in the seamless identification of travelers, without halts or interruptions of the train movement.

2) Enabling citizens of EU and non-EU countries to manage their passports through a secure digital platform accessible via mobile devices, simultaneously ensuring compliance with EU laws and regulations.

3) Ensuring sufficient protection and authentication of travelers’ identities, conducted through a various array of verification methods.

4) Providing vehicle scanning mechanisms at border control points without interrupting or halting train movements, which would detect illegal substances, vehicle modifications and unauthorized immigrants, fortifying border security measures.

It is important to note that the train pilot is split into two different parts: (1) passenger border crossing by train and (2) cargo train scanning. The first scenario is overseen by UIC and, in line with the current planning, the sequence of the first part of the train pilot is as follows:

1) Traveler pre-registers his/her journey in the ODYSSEUS platform,

2) BGAs prepare to receive the train and conduct the ID verification,

3) upon arrival, if a traveler is pre-registered in the ODYSSEUS platform, their ID is subject to verification,

4) in instances where there is an unsuccessful verification, a physical check is conducted,

5) collected data is then transmitted to the risk assessment component for further assessment and processing.

The upcoming rail passenger pilot will serve as a trial for technologies developed within the ODYSSEUS project and an in-situ evaluation opportunity for the technological capabilities in the sphere of for passenger identification. ODYSSEUS technologies will be tested in a real-life scenario at the Schengen border, and it is expected that with the help of these technologies the BGAs will be able to perform the border checks faster and easier for everyone.

The second rail pilot focuses solely on non-obtrusive and safe X-Ray technology for train wagon cargo scanning. It is conducted in a closed environment by consortium partner RAPISCAN Ltd. The key part of the developed technology is to enhance the border control of cargo trains in order to detect illicit goods faster and more reliably while the train is on the move.

At the end of the project the developed technology may be fully operationalized during the trains’ journey, and it may remain uninterrupted by halts. Preliminary key performance indicators (KPIs) indicate an anticipated reduction in costs for both BGAs and citizens travelling across the border, with an improvement in the user experience on both sides. Anticipated within the framework of the project is a very high level of accuracy in identity verification, as well as significant reduction of the time required for said verification process. The evaluation during those two train pilots will demonstrate the effectiveness of ODYSSEUS in facilitating cross-border travel for pre-registered travelers and showcase the technology potential for enhancing the overall control capabilities of BGAs.

## Railway challenges beyond security technologies for secure borders

While the tools being developed in the ODYSSEUS project address certain rail border crossing security concerns, there remain other challenges. Mainly, the challenges associated with refugee/migrant crises. The UIC Refugee Task Force published a guidance document on different organizational measures, such as how to organize “welcome zones” or deal with an increase in left luggage ( UIC, 2022). Another challenge which may be partly addressed by ODYSSEUS technology is that of the threat of trafficking in human beings (THB). One aspect in the fight against THB is related to identity documents, and the ODYSSEUS project is addressing this aspect. It is therefore important to underline that even though the technology developed in the ODYSSEUS project may serve as a seamless passenger transport enabler in railways, passengers may still be susceptible to various threats. Technological advancements contribute significantly to enhancing security and efficiency; however, it is essential to recognize that a comprehensive approach is required. Factors such as cybersecurity, potential misuse of data, and unforeseen security challenges must be continuously addressed to ensure the holistic safety and well-being of passengers in the face of evolving threats. The technology should be seen as a valuable tool within a broader strategy aimed at providing a secure and seamless travel experience.

Overcrowding on trains and stations, including on platforms, can result from influx caused by refugees. This may provide a risk in the event of an emergency (fire safety and emergency routes). The “welcome” zones should be arranged with security in mind as they might also add to station congestion. Ukrainian migration crisis has demonstrated that major cities and capitals are the most sought-after locations for refugees. Due to extreme congestion brought on by the massive number of refugees, the border train stations struggle to operate normally. Violations of the house rules, as well as occasionally violent clashes between migrants/refugees, may affect the objective and subjective security of those who are at railway premises.

Weary from hours of travel and exposed to stressful factors, refugees might not be as conscious of their possessions. Every time forgotten luggage is discovered on railway property, the proper procedures must be implemented. After being inspected for potentially hazardous contents, left bags must be turned over to the lost property office or confiscated by the appropriate authorities. Unattended luggage should never be disregarded because it could pose a real threat. Furthermore, trespassing on railway tracks can also be an associated problem that may further hinder rail operations.

In situations involving large numbers of people, refugees, and uncertainty, irregular migrants may take advantage of the circumstances. Even if illegal migration has not been discussed in regard to Ukrainian refugees, this does not preclude the phenomena from occurring. The obligation of railway companies is restricted to notifying the relevant authorities of any suspicious circumstances or incidents involving suspected illegal migration.

Vulnerability of Ukrainian migrants to human trafficking may also occur. It is estimated that over 7,000 people annually become a victim of human trafficking in the EU ( European Parliament, 2021). Most of the cases concern either sexual exploitation or forced labor ( European Commission, 2023b). There is also a visible increase of suspected traffickers that operate in the EU. Railway stations may serve as the hub for human trafficking due to their nature. Vivid and crowded places allow criminals to blend in and remain anonymous, thus giving opportunities to exploit vulnerable individuals. The transient nature of the railway station makes it challenging to monitor and track such activities or potential victims. Countermeasures often require targeted strategies, for example – informative brochures, constant audio announcements and trained staff. Railway undertakings and station managers must be vigilant about the potential for incidents to transpire in railway zones and collaborate with relevant state agencies accordingly.

When dealing with refugee influx in rail operations, the key challenge involves effective crisis and crowd management. Addressing afore mentioned issues requires optimal strategies that are efficiently backed up by state-of-the-art technologies. Transposing these challenges into the railway cross-border area needs an all-round approach. Mitigating such threats involves legislative measures at the state level, effective border management by BGAs, customs and other immigration enforcement authorities and the integration of advanced technology for efficient border control.

## Potential vulnerabilities regarding the passenger rights

Whether the cross-border movement is planned or forced by external factors (e.g., war), the passenger is affected both by security measures implemented on the border premises and normative measures that stipulate the border passing process. Sources of vulnerabilities are in constant increase in the times of rapid technological advancements, increased interconnectivity and mobility, as well as globalization. In the times of rapid securitization of the borders, it is important to also reflect how this Foucauldian *Apparatus* ( Agamben, 2009) of control is affecting the autonomy, freedom, and security, mediating the relationship between individuals and authorities. The *Apparatus* in this context refers to the network of technologies, legal frameworks and other state practices that regulate EU cross-border travel. To prevent the *Apparatus* from overstepping, a harmonious balance between security measures and fundamental rights is needed, to ensure that travellers' autonomy is preserved even amid heightened securitization.

According to European Union Agency for Fundamental Rights (2020) survey on awareness of EU data protection rules and the GDPR, 55% of respondents are afraid that fraudsters or criminals will access their personal information and 30% are concerned about information being secretly accessed by foreign governments or corporations. It is critical for the actors that are orchestrating the cross-border operation to interplay individual data security and stray from any potential data misuse. Stringent security measures during the cross-border check also need to respect travelers’ fundamental rights, including the right to information.

Considering the European diversity in different languages and cultures, the procedure should be clearly conveyed via proper signaling signs and explained if data handling is unclear to the passenger – especially when it involves advanced technology like biometric analysis which is proposed in ODYSSEUS. Additionally, BGA officers involved in the cross-border check should provide support in case of scenarios like passenger crime report, theft from a person, detection of human trafficking. The officers involved should be well-versed in passenger rights regulations, ensuring that travellers receive appropriate support when faced with these unusual circumstances.

There are several factors that need to be discussed during the assessment of the current state of EU cross-border travel by train. One of the challenges for rail in this context stems from the fact that existing data, for example BGAs’ data, often combines land vehicle and railway transportation when addressing cross border movements, leading to combined “land” datasets ( Straż Graniczna, 2024). As a result, data that is available may be ambiguous, making it difficult to draw precise conclusions on e.g. migrant movements by rail, number of law-breaking incidents, types of crime, etc. It hinders the ability to draw precise conclusions on cross-border movements in railway transportation sphere. Additionally, this data often cannot be supplemented by private or state carriers, as it is often subject to trade secret or considered as sensitive data.

Another challenging factor that is currently coming into play in railway operations is the complexity of ensuring security and integrity of border operations. Understanding the threats and mitigating them is paramount for securing an efficient transportation environment. During the uncertain times like refugee crises, a sudden increase may cause operational disruptions which need to be addressed immediately or in a short notice. When dealing with refugee influx in rail operations, the key challenge involves effective crisis and crowd management. Addressing afore mentioned issues requires optimal strategies that are efficiently backed up by state-of-the-art technologies. Transposing these challenges into the railway cross-border area needs an all-round approach. Mitigating such threats involves legislative measures at the state level, effective border management by customs or other immigration enforcement and the integration of advanced technology for efficient border control.

The complicated interplay between geopolitics and securitization affects many aspects of rail border security, such as procedures and policies. For example, the attitude towards migrants of any given State greatly impacts their border policies. As Bello (2017) discussed, European states may be more prejudiced toward migrants if the nation narrations of a particular country are creating leeway for that. Along these lines, views towards a given refugee group may also change over time. For example, in April 2022, Poland had the biggest approval rate of Ukrainian migrants (84%) in Europe, whereas two years later, according to the DG COMM’s Public Opinion Monitoring Unit data from February 2024, the approval rate dropped down to 60%, the lowest score in Europe ( European Parliament, 2024).

## Conclusion

ODYSSEUS as a facilitator of seamless cross-border travel experiences, holds the potential to create positive impacts on rail transportation if integrated as a tool for end-users. Its implementation can address several challenges and enhance the efficiency of cross-border rail travel. From an ethical standpoint the ODYSSEUS technologies may be viewed as invasive, particularly from the perspective of vulnerable travelers such as migrants or refugees, who ultimately may feel like *Homo Sacer* – individuals subject to arbitrary control. This raises questions about how these technologies affect the autonomy and dignity of individuals. *Apparatus* of control, embodied by these screening technologies, must be carefully managed to prevent overreach. This includes regular passengers who willingly consent for screening, as well as more vulnerable groups, such as minors, migrants, and war refugees. The ethical challenge posed by the screening technologies can be mitigated. For instance, clear signaling and communication of the screening process by state agencies can enhance transparency and build trust among travelers. Striking a balance between security needs and respecting individual rights is crucial, and these proactive steps can contribute to addressing the ethical concerns associated with the application of such screening technologies. In the evolving socio-economic context and the growing securitization of borders, this *dispositif* of technology and surveillance must be balanced in terms of security need and fundamental rights of EU and non-EU travellers. By adjusting to these circumstances – whether geopolitical, social or economic – ODYSSEUS solution may remain relevant, adaptable and they can uphold the ethical standards essential to those sensitive and critical concepts. By adjusting to these factors, the ODYSSEUS technological solutions better suit changing dynamics, ensuring the project's relevance in terms of effectiveness and applicability to the real-life border crossing scenarios.

## Limitations and perspectives

This work-in-progress paper is meant to be a first critical reflection of the railway-related challenges as well as a preliminary contribution to the project’s dissemination strategy. As such is has a number of limitations characteristic to a reflective paper. The current work will be expanded into a research article once the ODYSSEUS train pilot is completed. This will involve the development of a questionnaire-based methodology to collect data from pilot participants. During the pilot, UIC together with other project partners will evaluate the feedback provided by the participants, assess the solution's usability and user acceptability and confirm that the intended KPIs have been effectively reached. The data will allow a qualitative and quantitative analysis and lead to novel results in a more developed version of this this paper. After train pilot assessment, UIC will continue to contribute to the dissemination of the project results and facilitate the results exploitation in its European region and among the railway sector.

## Data Availability

No data are associated with this article.
