# Supplementary material for: Railway security checks at the border: between intrusive security technologies and fundamental traveller rights
Source: Open Res Eur. 2024 Oct 15;4:138. Originally published 2024 Jul 9. [Version 2] doi: 10.12688/openreseurope.17727.2 (PMC11499734; doi:10.12688/openreseurope.17727.2)
Supplement: Peer review history

# Reviewer report: Reviewer response for version 2

**DOI:** 10.21956/openreseurope.20011.r45175

**Published:** 2024-10-23

**Authors:** Ridwan Arifin

**Affiliations:** 1 Immigration Polytechnic, University of Indonesia, Depok, Indonesia; 2 Immigration Administration, immigration Polytechnic, Depok, west Java, Indonesia

Thank you for your insightful and thorough revisions to the manuscript. I appreciate the time and effort you’ve taken to clarify the methodology section and elaborate on the scope and intention of the paper. Your detailed explanation about the nature of the manuscript as a discussion article rather than an empirical study is particularly helpful, especially in distinguishing its focus in relation to the EU-funded ODYSSEUS project. I also appreciate the enhancements you've made in response to the journal’s format requirements and the integration of theoretical perspectives, particularly regarding border securitization and the principle of free movement. The addition of references to Bentham’s Panopticon and Foucault's ‘dispositif’ brings greater depth to the discussion, offering a richer theoretical context for understanding the implications of security measures on individual rights and freedoms. Your acknowledgment of the paper's limitations and the reframing of it as a preliminary exploration rather than a research article further strengthens the clarity of its contribution. This makes the paper more focused and aligns well with its role in informing the future railway pilot trial in the ODYSSEUS project. Given these thoughtful revisions and the clear articulation of the paper’s objectives and scope, I agree that the manuscript is now ready for indexing. Thank you again for addressing the feedback with such careful consideration.

Is the study design appropriate and does the work have academic merit?

Partly

Is the work clearly and accurately presented and does it cite the current literature?

Partly

If applicable, is the statistical analysis and its interpretation appropriate?

Not applicable

Are all the source data underlying the results available to ensure full reproducibility?

Yes

Are the conclusions drawn adequately supported by the results?

Partly

Are sufficient details of methods and analysis provided to allow replication by others?

Partly

Reviewer Expertise:

International Migration and Border Studies, Strategic and Global Studies, Border Security Studies

I confirm that I have read this submission and believe that I have an appropriate level of expertise to confirm that it is of an acceptable scientific standard.

# Reviewer report: Reviewer response for version 2

**DOI:** 10.21956/openreseurope.20011.r45174

**Published:** 2024-10-17

**Authors:** Terence Michael Garrett

**Affiliations:** 1 The University of Texas Rio Grande Valley, Brownsville, Texas, USA

The authors have done well addressing my concerns. This article is now more academically sound and the changes made are satisfactory.

Is the study design appropriate and does the work have academic merit?

Partly

Is the work clearly and accurately presented and does it cite the current literature?

Partly

If applicable, is the statistical analysis and its interpretation appropriate?

Not applicable

Are all the source data underlying the results available to ensure full reproducibility?

Yes

Are the conclusions drawn adequately supported by the results?

Partly

Are sufficient details of methods and analysis provided to allow replication by others?

No

Reviewer Expertise:

PhD in Political Science. Specialties: Border Security (Insecurity); Migration Policy; Public Administration

I confirm that I have read this submission and believe that I have an appropriate level of expertise to confirm that it is of an acceptable scientific standard.

# Reviewer report: Reviewer response for version 1

**DOI:** 10.21956/openreseurope.19163.r42692

**Published:** 2024-08-16

**Authors:** Ridwan Arifin

**Affiliations:** 1 Immigration Polytechnic, University of Indonesia, Depok, Indonesia; 2 Immigration Administration, immigration Polytechnic, Depok, west Java, Indonesia

The manuscript presents a strong background and a clear identification of the issues at hand in the Abstract, which effectively sets the stage for the study. However, there are significant gaps in the methodology section, where the types of methodologies employed, as well as the processes of data collection and analysis, are not sufficiently detailed. This lack of clarity makes it difficult to assess the robustness of the research design and the validity of the findings.

 In terms of results, the manuscript fails to demonstrate the novelty of the theoretical or conceptual framework related to EU border security control at railway security checks. The analysis does not adequately address the critical tension between the securitization of borders and the principle of free movement, particularly in the context of significant contemporary issues such as the management of migrants and refugees. This is a crucial oversight, as the balance between security measures and human rights is central to the ongoing debate in EU policy circles.

 The conclusion section is also lacking in depth and fails to provide a comprehensive analysis of how the ODYSSEUS framework can facilitate and balance the securitization of borders, the principle of free movement, and the issues surrounding migrants and refugees at railway stations, platforms, and on trains. The discussion does not offer solutions to these pressing challenges, leaving the reader with unanswered questions.

 Moreover, the manuscript is underpinned by a very limited theoretical framework, with most citations referencing official reports from EU bodies such as Frontex and the UIC. There is a noticeable lack of engagement with academic literature, and several paragraphs present arguments that are not adequately supported by references or sources. This weakens the overall argument and reduces the manuscript's contribution to the scholarly discourse on EU border security and human rights.

 Overall, while the manuscript has potential, it requires significant revisions to clarify the methodology, enhance the analysis, and strengthen the theoretical foundation through more extensive engagement with relevant academic literature.

Is the study design appropriate and does the work have academic merit?

Partly

Is the work clearly and accurately presented and does it cite the current literature?

Partly

If applicable, is the statistical analysis and its interpretation appropriate?

Not applicable

Are all the source data underlying the results available to ensure full reproducibility?

Yes

Are the conclusions drawn adequately supported by the results?

Partly

Are sufficient details of methods and analysis provided to allow replication by others?

Partly

Reviewer Expertise:

International Migration and Border Studies, Strategic and Global Studies, Border Security Studies

I confirm that I have read this submission and believe that I have an appropriate level of expertise to confirm that it is of an acceptable scientific standard, however I have significant reservations, as outlined above.

# Response

**Published:** 2024-09-24

**Authors:** Kacper Kubrak

**Affiliations:** Security Division, International Union of Railways, Paris, Île-de-France, France

We appreciate your review of the manuscript and your comments.

 We acknowledge your concerns regarding the methodology section, data collection, etc., and we have made significant revisions to enhance clarity concerning these issues.

 The journal required the abstract to include the classical sections of a research article (Background, Methods, Results, Conclusion) and these headings can be misleading since our paper was not intended as an empirical study, thus we have not collected and analyzed data in line with a replicable methodology.

 The paper was never designed as a research article. Furthermore, it was never the paper’s objective to prove a new theoretical or conceptual framework related to EU border security.

 Our paper was proposed as a preliminary discussion article (work-in-progress) in the context of the future railway pilot trial planned in the European project ODYSSEUS which funds this work.

 The aim of the paper and its very focused scope is now better explained in a dedicated section. In line with your comments, we recognize the importance of engaging with a broader range of academic literature and have integrated several new references and concepts which were suggested by the other referee.

 Specifically, we now address the tension between border securitization and the principle of free movement more comprehensively, incorporating more theoretical insights. For example, our discussion on the balance between cross-border traveller rights and the constantly expanding securitization of borders was broadened through engagement with more relevant academic literature such as modern manifestations of Bentham's Panopticon.

 We also framed our discussion around Foucault's notion of the ‘ *dispositif*’, and we discuss how these control mechanisms impact individual rights and freedoms, reinforcing the need to balance security measures with the protection of human dignity.

 Finally, we have made the paper's limitations more explicit, emphasizing that this work is a preliminary reflective exploration of the challenges associated with railway security in cross-border settings, approached through discussion rather than empirical research.

 We believe these revisions address your concerns on the manuscript, especially while considering the focused scope of our paper which needs to be contained in the framework of the EU-funded project ODYSSEUS.

# Reviewer report: Reviewer response for version 1

**DOI:** 10.21956/openreseurope.19163.r42340

**Published:** 2024-08-08

**Authors:** Terence Michael Garrett

**Affiliations:** 1 The University of Texas Rio Grande Valley, Brownsville, Texas, USA

The manuscript is essentially lacking a research design. It is largely without an identifiable theoretical framework and there is no reflection on methodology. 

 The authors should consider the implications of the application of biometrics and its consequences for society. Biometrics, for example, is part and parcel to Jeremy Bentham's panopticon(Cruz  et al.,1996)(Ref-3), also analyzed by scholars such as Michel Foucault(Foucault et al.,1977)(Ref-9). There is an extensive literature concerning the impact of the panopticon and the authors might tap into it.

 Additionally, they may want to address those for whom are the target of the panopticon (or "banopticon" see Didier Bigo's work)(Tsoukala  et al.,2006)(Ref-4). There is an extensive analytical literature on *dispositif *(Foucault again)(Foucault et al.,2008)(Ref-6), and apparatuses (Giorgio, et al.,2009)(Ref-8). These biometric measures impact the *homo sacer* (Nikolopoulou et al.,1995)(Ref-1) - all designed to control or expel migrants and potential "terrorists" - also known as the process of "othering."

 Furthermore, a theoretical framework could reflect the neoliberal impulse to prevent or control asylum seekers and refugees (the so-called [reduction of] "illegal movements of people and goods across EU borders") from the global south entering the comparatively wealthier global north countries so that " [allowing for the [facilitation of] traveling for citizens while protecting fundamental rights of travelers." Wendy Brown(Brown et al.,2019)(Ref-5) and Harsha Walia's (Walia et al., 2021)(Ref-7) work might be helpful.

 In short, there are numerous ways to engage academically this important topic - well beyond what is discussed here. The authors should consider doing so.

Is the study design appropriate and does the work have academic merit?

Partly

Is the work clearly and accurately presented and does it cite the current literature?

Partly

If applicable, is the statistical analysis and its interpretation appropriate?

Not applicable

Are all the source data underlying the results available to ensure full reproducibility?

Yes

Are the conclusions drawn adequately supported by the results?

Partly

Are sufficient details of methods and analysis provided to allow replication by others?

No

Reviewer Expertise:

PhD in Political Science. Specialties: Border Security (Insecurity); Migration Policy; Public Administration

I confirm that I have read this submission and believe that I have an appropriate level of expertise to confirm that it is of an acceptable scientific standard, however I have significant reservations, as outlined above.

## References

[1] : Homo Sacer: Sovereign Power and Bare Life. *SubStance* .2000;29(3) : 10.2307/3685567 10.2307/3685567

[2] : Simulacra and Simulation.1995; 10.3998/mpub.9904 10.3998/mpub.9904

[3] : J. Bentham. The Panopticon Writings, Edited and Introduced by Miran Bozovic Verso. Londres-Nueva York, 1995, 158 pp. *Persona y Derecho* .1970; 10.15581/011.32443 274-279 10.15581/011.32443

[4] : Understanding (in)security.20083125: 10.4324/9780203926765.ch1 10.4324/9780203926765.ch1

[5] : In the Ruins of Neoliberalism.2019; 10.7312/brow19384 10.7312/brow19384

[6] The Birth of Biopolitics.2008; 10.1057/9780230594180 10.1057/9780230594180

[7] : Border and rule: Global migration, capitalism, and the rise of racist nationalism. *https://www.haymarketbooks.org/books/1553-border-and-rule* .2021;

[8] : "What Is an Apparatus?" and Other Essays. *https://www.sup.org/books/title/?id* .2009;

[9] : DISCIPLINE AND PUNISH The Birth of the Prison. *https://monoskop.org/images/4/43/Foucault_Michel_Discipline_and_Punish_The_Birth_of_the_Prison_1977_1995.pdf* .1977; 10.2307/797132. 10.2307/797132.

# Response

**Published:** 2024-09-24

**Authors:** Kacper Kubrak

**Affiliations:** Security Division, International Union of Railways, Paris, Île-de-France, France

We appreciate your thorough review and insightful suggestions. We improved the paper based on your comments while keeping it within its defined scope. This paper is not an empirical study but a critical reflection on the specific security challenges tied to the railway context, particularly within the European ODYSSEUS project, which funds this research. Moreover, the paper was never meant to be a thorough analysis of biometrics or an analysis of their consequences for society in general. While we acknowledge the importance of concepts like “panopticon”, “banopticon” “simulacrum” and “apparatus”, an in-depth discussion of all these theories would extend the scope of the paper far beyond the ambition and the resources of the project. Therefore, we clarified the sections which explain the aim of the paper, as well as its limitations, and we revised some of the subtitles to improve clarity. That said, based on your recommendations, we have significantly enriched the theoretical framework with most of the concepts and theories that you suggested. Seven out of the nine suggested references were integrated. For example, the introduction is now more academically engaged and incorporates a reflection on *Homo Sacer*, contextualizing it within modern EU cross-border dynamics. Further, in the newly added section, "Challenges in European Border Management," we introduced the theoretical frameworks drawn from the works of Harsha, Brown, Foucault, Bentham, and Bigo. We have used these perspectives to critically examine the *apparatus* of control ( *dispositif*) that modern border management represents. Furthermore, in light of these frameworks, the conclusion was strengthened, emphasizing the need to balance security with the protection of fundamental rights. We discussed the risks of technological overreach and surveillance, drawing parallels with Bentham’s *Panopticon*, and underscored that security innovations cannot be allowed to undermine passenger autonomy or human dignity. We trust that these revisions address your concerns, while considering the focused scope of our paper which needs to be contained in the framework of the EU-funded project ODYSSEUS.
